# Out‐of‐hospital cardiac arrests in Victoria, 2003–2022: retrospective analysis of Victorian Ambulance Cardiac Arrest Registry data

**DOI:** 10.5694/mja2.52532

**Published:** 2024-11-18

**Authors:** Emily Nehme, David Anderson, Ross Salathiel, Anthony Carlyon, Dion Stub, Peter A Cameron, Andrew Wilson, Sile Smith, John J McNeil, Ziad Nehme

**Affiliations:** ^1^ Centre for Research and Evaluation Ambulance Victoria Melbourne VIC; ^2^ Monash University Melbourne VIC; ^3^ Alfred Health Melbourne VIC; ^4^ Ambulance Victoria Melbourne VIC; ^5^ St Vincent's Heart Centre St Vincent's Health Australia Melbourne VIC; ^6^ Royal Children's Hospital Melbourne VIC

**Keywords:** Emergency services, medical, Resuscitation, Emergency medical services, Registries

## Abstract

**Objectives:**

To examine changes in out‐of‐hospital cardiac arrest (OHCA) characteristics and outcomes during 2003–2022, and 12‐month outcomes for people who experienced OHCA during 1 January 2010 – 30 June 2022.

**Study design:**

Retrospective observational study; analysis of Victorian Ambulance Cardiac Arrest Registry (VACAR) data.

**Setting, participants:**

OHCA events in Victoria not witnessed by emergency medical services personnel, 1 January 2003 – 31 December 2022.

**Main outcome measures:**

Crude and age‐standardised annual OHCA incidence rates; survival to hospital discharge.

**Results:**

Of 102 592 OHCA events included in our analysis, 67 756 were in men (66.3%). The age‐standardised incidence did not change significantly across the study period (2003: 89.1 cases, 2022: 91.2 cases per 100 000 population; for trend: *P* = 0.50). The proportion of OHCA cases with attempted resuscitation by emergency medical services in which bystanders attempted cardio‐pulmonary resuscitation increased from 40.3% in 2003/2004 to 72.2% in 2021/2022, and that of public access defibrillation from 0.9% to 16.1%. In the Utstein comparator group (witnessed OHCA events in which the initial cardiac rhythm was ventricular fibrillation or ventricular tachycardia, with attempted resuscitation by emergency medical services), the odds of survival to hospital discharge increased during 2003–2022 (adjusted odds ratio (aOR), 3.08; 95% confidence interval [CI], 2.22–4.27); however, the odds of survival was greater than in 2012 only in 2018 (aOR, 1.37; 95% CI, 1.04–1.80) and 2019 (aOR, 1.68; 95% CI, 1.28–2.21). The COVID‐19 pandemic was associated with reduced odds of survival (aOR, 0.63; 95% CI, 0.54–0.74). Of 3161 people who survived OHCA and participated in 12‐month follow‐up, 1218 (38.5%) reported full health according to the EQ‐5D.

**Conclusion:**

Utstein survival to hospital discharge increased threefold during 2003–2022, and the proportions of cases in which bystanders provided cardio‐pulmonary resuscitation or public access defibrillation increased. The COVID‐19 pandemic was associated with a substantial reduction in survival, and new strategies are needed to improve outcomes.

**The known**: Survival after out‐of‐hospital cardiac arrest depends on the “chain of survival”. The Victorian Ambulance Cardiac Arrest Registry is uniquely valuable for assessing long term changes in out‐of‐hospital cardiac arrest characteristics and patient outcomes.

**The new**: In Victoria, survival after bystander‐witnessed out‐of‐hospital cardiac arrest events with initially shockable rhythms increased threefold during 2003–2022, and was associated with more frequent cardiopulmonary resuscitation and public access defibrillation by bystanders. Survival gains since 2012, however, were evident only in 2018 and 2019. Survival declined significantly during the COVID‐19 pandemic.

**The implications**: New strategies are needed to further improve survival after out‐of‐hospital cardiac arrest.

Sudden cardiac arrest is a major public health challenge.^1^ Survival to hospital discharge for adults who experience out‐of‐hospital cardiac arrest (OHCA) and receive cardiopulmonary resuscitation (CPR) is estimated to be 8.8%.^2^ Survival depends on the “chain of survival”, particularly its first three links: immediate recognition of cardiac arrest and activation of emergency medical services (EMS), early CPR, and early defibrillation.^1^ Initiatives that enhance these links are crucial for improving outcomes for people who experience OHCA.

Local and overseas efforts to improve survival after OHCA have focused on encouraging CPR and defibrillation by bystanders.^3^, ^4^, ^5^, ^6^ In Victoria, changing EMS telephone CPR instructions to applying 400 compressions before ventilation was associated with increased bystander provision of CPR^7^ and improved survival.^8^ Similarly, 30‐day OHCA survival in Denmark tripled after national initiatives that encouraged bystander CPR.^4^ Recently, smartphone applications that activate community responders have also been associated with increased bystander intervention rates.^9^, ^10^

The Global Resuscitation Alliance has recommended establishing a cardiac arrest registry as the first step to improving OHCA survival, as a registry is the essence of measurement, and continuous measurement is fundamental to improvement.^11^ The Victorian Ambulance Cardiac Arrest Registry (VACAR), established in 1999, monitors OHCA events in Victoria; since 2011, it has also captured data on 12‐month outcomes for people who survive OHCA. As one of the oldest cardiac arrest registries in the world, the VACAR is uniquely placed to assess the impact of quality improvement initiatives and long term changes in outcomes. In this article, we report changes in OHCA characteristics and outcomes in Victoria during 2003–2022, and 12‐month outcomes for people who experienced OHCA during 1 January 2010 – 30 June 2022.

## Methods

We conducted a retrospective observational study of OHCA cases attended by emergency medical services in Victoria during 1 January 2003 – 31 December 2022. We included all OHCA cases, regardless of patient age and aetiology, but excluded OHCAs witnessed by EMS personnel, as they comprise a specific OHCA population with different predictors of survival. We also excluded cases in which witness status was unknown.

### Setting

The population of Victoria is about 6.6 million people, about four million of whom live in Melbourne. A single EMS, Ambulance Victoria, operates across the state. Suspected OHCA events identified during emergency Triple Zero (000) calls lead to telephone CPR instructions for the caller, and the dual dispatch of advanced life support and mobile intensive care ambulance paramedics. In Melbourne and some regional areas, basic life support‐trained firefighters and community emergency volunteers can be dispatched as first responders. In 2018, Ambulance Victoria launched a volunteer responder smartphone application (GoodSAM; https://www.ambulance.vic.gov.au/goodsam) and made an automated external defibrillator (AED) registry publicly available (https://registermyaed.ambulance.vic.gov.au), both of which are integrated with the dispatch system. Registered volunteers within 500 m of a suspected OHCA in Melbourne or within 5 km in regional areas are prompted to attend to provide CPR or to retrieve a public AED.

Paramedic treatment guidelines follow the recommendations of the Australian and New Zealand Committee on Resuscitation.^12^ In 2019, Ambulance Victoria implemented a resuscitation quality improvement program in which paramedics were trained in high performance CPR and team choreography, real‐time CPR feedback, and post‐event debriefing.^13^

### Data sources

The VACAR is a population‐based register of all OHCA events attended by EMS in Victoria.^8^ Potential OHCA cases are identified by a sensitive electronic search algorithm of patient care record data stored in a data warehouse. After manual review by trained staff, confirmed OHCA cases are entered into the registry according to Utstein recommendations.^14^ Hospital survival is ascertained in receiving hospitals data and validated by Victorian Registry of Births, Deaths and Marriages data. Since 2011, 12‐month telephone follow‐up interviews with adults who survive OHCA have assessed their health‐related quality of life, functional recovery, and residential and return‐to‐work status.^15^ Functional recovery is measured with the Glasgow Outcome Scale–Extended,^16^ and health‐related quality of life with the EQ‐5D^17^ (to June 2020, the EQ‐5D‐3L; from July 2020 the EQ‐5D‐5L). Follow‐up data for this study were available to 30 June 2022.

### Definitions

We defined “resuscitation attempted by EMS” as any CPR or defibrillation attempt by paramedics or first responders, regardless of duration, or attempted defibrillation with a public AED. Arrest aetiology was defined according to Utstein recommendations, and was presumed to be cardiac unless otherwise clearly documented.^14^ The Utstein comparator group included bystander‐witnessed arrests in which the initial cardiac rhythm was ventricular fibrillation or ventricular tachycardia, and resuscitation was attempted by EMS personnel. An EQ‐5D index score of one indicated full health at twelve months.

### Statistical analyses

Baseline characteristics for all OHCA cases and for cases in which resuscitation was attempted are separately reported as two‐year groups. We summarise categorical data as frequencies and proportions, normally distributed continuous data as means with standard deviations (SDs), and skewed continuous data as medians with interquartile ranges (IQRs). We calculated crude and age‐standardised annual OHCA incidence rates using Australian Bureau of Statistics Victorian population estimates for 2001 (5‐year age groups).^18^ Long term change was assessed in linear regression analyses.

For the Utstein comparator group, we estimated the annual adjusted odds of survival to hospital discharge (reference year: 2012) in a multivariable logistic regression analysis adjusted for study year, age, gender, aetiology (presumed cardiac *v* trauma or hanging, overdose or poisoning, or other), arrest location (public location *v* private residence, aged care facility, or other), and arrest geographic location (metropolitan *v* regional). We did not adjust our analyses for bystander CPR, public access defibrillation, or EMS response time, given the introduction of improvement strategies in these areas during the study period. In a separate analysis of the association of study year with survival, we added year as a continuous term and a term to account for the coronavirus disease 2019 (COVID‐19) pandemic (from 16 March 2020).

We also constructed two multivariable logistic regression models to examine the odds of receiving bystander CPR or public access defibrillation in cases with an initial rhythm of ventricular fibrillation or ventricular tachycardia (initially shockable). These models were adjusted for study year, age, gender, aetiology, arrest location, witness status (arrest witnessed *v* not witnessed), and geographic location of arrest. We report adjusted odds ratios (aORs) with 95% confidence intervals (CIs). Statistical analyses were performed in Stata 18.0; *P* < 0.05 was deemed statistically significant.

### Ethics approval

The VACAR has ethics approval from the Monash University Human Research Ethics Committee (21046), as well as from more than 100 participating hospitals across Victoria.

## Results

A total of 113 108 OHCA events were recorded in Victoria during 2003–2022; after excluding 9436 EMS‐witnessed arrests and 1080 arrests with missing witness status, 102 592 OHCA events were included in our analysis, of which 67 756 were in men (66.3%).

### Incidence of out‐of‐hospital cardiac arrest

Crude OHCA incidence rose from 90.6 cases per 100 000 population in 2003 to 107.4 per 100 000 population in 2022 (for trend: *P* = 0.001). The age‐standardised incidence did not change significantly across this period (2003: 89.1 cases per 100 000 population; 2022: 91.2 cases per 100 000 population; for trend: *P* = 0.50), although it increased during 2020–2022 after declining during 2003–2013 (Box 1).

Box 1 Crude and age‐standardised incidence of out‐of‐hospital cardiac arrest, Victoria, 2003–2022*

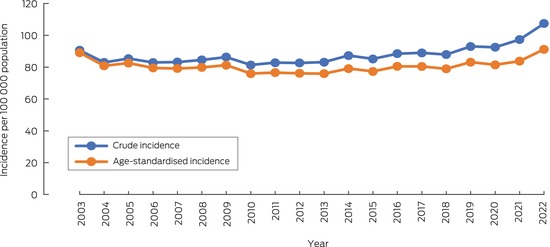

* Excludes emergency medical service‐witnessed events. The data underlying this graph are included in the Supporting Information, table 1.

### Survival to hospital discharge after out‐of‐hospital cardiac arrest

From 2003, survival steadily increased to peaks in 2019 of 38.3% for cases in people with initially shockable rhythms (240 of 626 cases) and 42.8% in the Utstein group (207 of 484), then declined respectively to 27.2% (162 of 596 cases) and 31.4% (141 of 459) in 2020. The EMS resuscitation quality improvement program was launched in 2019; the COVID‐19 pandemic was declared in 2020 (Box 2). The incidence of survival to hospital discharge increased from 2.2 per 100 000 population in 2003 to 4.6 per 100 000 population in 2019, then declined to 4.0 per 100 000 population in 2022 (for trend: *P* = 0.001).

Box 2 Survival to hospital discharge after out‐of‐hospital cardiac arrest with attempted resuscitation by emergency medical services, Victoria, 2003–2022*

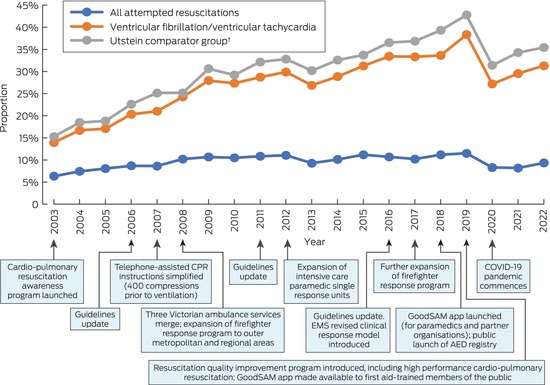

AED = automated external defibrillator; EMS = emergency medical services; COVID‐19 = coronavirus disease 2019; CPR = cardio‐pulmonary resuscitation.

* Excludes emergency medical service‐witnessed events. The data underlying this graph are included in the Supporting Information, table 2.

† Bystander‐witnessed arrests in which the initial cardiac rhythm was ventricular fibrillation or ventricular tachycardia, and resuscitation was attempted by emergency medical services.

The adjusted odds of survival to hospital discharge for people in the Utstein comparator group (reference year: 2012) increased steadily from 2003 to 2009, then plateaued during 2009–2017. The odds of survival during 2013–2022 was greater than in 2012 only in 2018 (aOR, 1.37; 95% CI, 1.04–1.80) and 2019 (aOR, 1.68; 95% CI, 1.28–2.21) (Box 3). The odds of survival were much higher than in 2003 in 2019 (aOR, 4.35; 95% CI, 3.13–6.04) and 2022 (aOR, 3.08; 95% CI, 2.22–4.27). In the analysis including year as a continuous term and adjusted for the COVID‐19 pandemic, the linear annual increase in odds of survival during 2003–2022 was statistically significant (aOR, 1.08; 95% CI, 1.06–1.09). The COVID‐19 pandemic was associated with reduced odds of survival (aOR, 0.63; 95% CI, 0.54–0.74).

Box 3 Survival to hospital discharge after out‐of‐hospital cardiac arrest, Victoria, 2003–2022, for cases in the Utstein comparator group: adjusted odds ratios with 95% confidence intervals (reference year: 2012)*

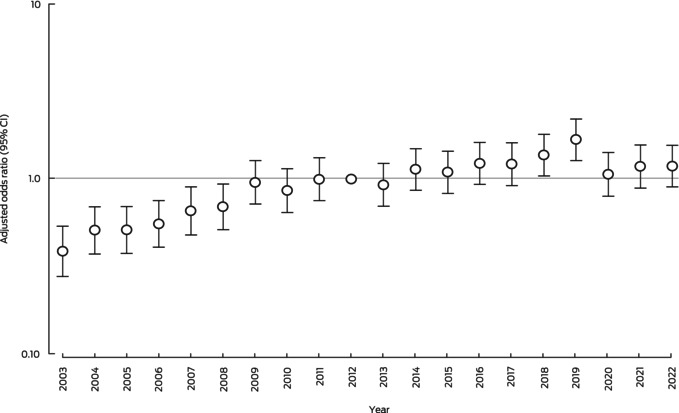

* Adjusted for study year, age, gender, aetiology (presumed cardiac *v* trauma or hanging, overdose or poisoning, or other), arrest location (public location *v* private residence, aged care facility, or other), and arrest geographic location (metropolitan *v* regional). The data underlying this graph are included in the Supporting Information, table 3.

### Out‐of‐hospital cardiac arrest event characteristics

#### Bystander cardiopulmonary resuscitation

The proportion of bystander‐witnessed OHCAs declined from 34.3% in 2003/2004 to 29.1% in 2021/2022; the proportion of all OHCAs with bystander CPR increased over the same period from 20.3% to 39.2% (Box 4, Box 5A). Among OHCA cases in which resuscitation was attempted by EMS personnel, the proportion with bystander CPR increased from 40.3% in 2003/2004 to 72.2% in 2021/2022 (Box 6, Box 5A). The adjusted odds of receiving bystander CPR were higher in 2022 than 2003 (aOR, 4.54; 95% CI, 3.99–5.17).

Box 4 Characteristics of out‐of‐hospital cardiac arrests, Victoria, 2003–2022, by two‐year period*

**Table:** 

Characteristic	Overall	2003/2004	2005/2006	2007/2008	2009/2010	2011/2012	2013/2014	2015/2016	2017/2018	2019/2020	2021/2022
Total	102 592	8670	8663	9019	9313	9391	9990	10 594	11 216	12 205	13 531
Age (years), mean (SD)^†^	64.4 (20.5)	64.0 (20.2)	63.9 (20.6)	64.4 (20.6)	64.2 (20.9)	64.0 (20.8)	64.5 (20.1)	64.1 (20.8)	64.2 (20.4)	64.6 (20.4)	65.9 (19.8)
Sex (men)^‡^	67 756 (66.3%)	5720 (66.5%)	5729 (66.6%)	5938 (66.3%)	6060 (65.2%)	6250 (66.7%)	6574 (66.0%)	7079 (67.0%)	7363 (65.8%)	8135 (66.8%)	8908 (66.0%)
Metropolitan location	70 591 (68.8%)	6203 (71.6%)	6184 (71.4%)	6386 (70.8%)	6442 (69.2%)	613 (68.3%)	6841 (68.5%)	7230 (68.3%)	7645 (68.2%)	8203 (67.2%)	9044 (66.8%)
Location											
Private residence	77 764 (75.8%)	6521 (75.2%)	6517 (75.2%)	6738 (74.7%)	6944 (74.6%)	6978 (74.3%)	7505 (75.1%)	8055 (76.0%)	8435 (75.2%)	9434 (77.3%)	10 637 (78.6%)
Aged care facility	7731 (7.5%)	503 (5.8%)	533 (6.2%)	655 (7.3%)	743 (8.0%)	709 (7.6%)	817 (8.2%)	836 (7.9%)	951 (8.5%)	988 (8.1%)	996 (7.4%)
Public location	15 129 (14.8%)	1464 (16.9%)	1419 (16.4%)	1460 (16.2%)	1452 (15.6%)	1531 (16.3%)	1471 (14.7%)	1513 (14.3%)	1618 (14.4%)	1528 (12.5%)	1673 (12.4%)
Other	1968 (1.9%)	182 (2.1%)	194 (2.2%)	166 (1.8%)	174 (1.9%)	173 (1.8%)	197 (2.0%)	190 (1.8%)	212 (1.9%)	255 (2.1%)	225 (1.7%)
Precipitating event											
Presumed cardiac	72 992 (71.2%)	6218 (71.7%)	6213 (71.7%)	6450 (71.5%)	6752 (72.5%)	6428 (68.5%)	7029 (70.4%)	7516 (71.0%)	8071 (72.0%)	8716 (71.4%)	9599 (70.9%)
Trauma/hanging	12 014 (11.7%)	994 (11.5%)	1051 (12.1%)	1054 (11.7%)	1149 (12.3%)	1149 (12.2%)	1202 (12.0%)	1261 (11.9%)	1303 (11.6%)	1393 (11.4%)	1458 (10.8%)
Overdose/poisoning	5157 (5.0%)	524 (6.0%)	437 (5.0%)	488 (5.4%)	441 (4.7%)	456 (4.9%)	493 (4.9%)	541 (5.1%)	595 (5.3%)	590 (4.8%)	592 (4.4%)
Other	12 429 (12.1%)	934 (10.8%)	962 (11.1%)	1027 (11.4%)	971 (10.4%)	1358 (14.5%)	1266 (12.7%)	1276 (12.0%)	1247 (11.1%)	1506 (12.3%)	1882 (13.9%)
EMS response time (min), median (IQR)^§^	8.2 (6.2–12.0)	7.0 (6.0–10.0)	8.0 (6.0–11.0)	8.3 (6.3–11.9)	8.6 (6.5–12.3)	8.7 (6.5–12.8)	8.5 (6.4–12.6)	8.1 (6.1–11.8)	7.9 (6.0–11.5)	8.2 (6.2–11.8)	9.0 (6.7–14.8)
Bystander‐witnessed	31 829 (31.0%)	2970 (34.3%)	2808 (32.4%)	2705 (30.0%)	3021 (32.4%)	3121 (33.2%)	3259 (32.6%)	3266 (30.8%)	3299 (29.4%)	3446 (28.2%)	3934 (29.1%)
Bystander CPR	34 517 (33.6%)	1758 (20.3%)	1586 (18.3%)	2099 (23.3%)	2689 (28.9%)	3476 (37.0%)	4168 (41.7%)	4273 (40.3%)	4337 (38.7%)	4829 (39.6%)	5302 (39.2%)
Initial rhythm^¶^											
Ventricular fibrillation/ventricular tachycardia	12 509 (12.2%)	1234 (14.3%)	1211 (14.0%)	1097 (12.2%)	1198 (12.9%)	1282 (13.7%)	1315 (13.2%)	1289 (12.2%)	1289 (11.5%)	1257 (10.3%)	1337 (9.9%)
Pulseless electrical activity	9962 (9.7%)	943 (10.9%)	917 (10.6%)	813 (9.1%)	841 (9.1%)	980 (10.5%)	1045 (10.5%)	1144 (10.8%)	1165 (10.4%)	1142 (9.4%)	972 (7.2%)
Asystole	78 496 (76.7%)	6383 (73.9%)	6464 (74.9%)	7047 (78.5%)	7225 (77.9%)	7069 (75.6%)	7585 (76.1%)	8134 (76.9%)	8614 (77.0%)	9487 (78.0%)	10 488 (77.6%)
Non‐shockable (unspecified)	1 320 (1.3%)	82 (1.0%)	33 (0.4%)	16 (0.2%)	13 (0.1%)	26 (0.3%)	27 (0.3%)	8 (0.1%)	120 (1.1%)	277 (2.3%)	718 (5.3%)
Attempted resuscitation	43 195 (42.1%)	3543 (40.9%)	3422 (39.5%)	3519 (39.0%)	3785 (40.6%)	4048 (43.1%)	4518 (45.2%)	4660 (44.0%)	5049 (45.0%)	5132 (42.1%)	5519 (40.8%)

CPR = cardiopulmonary resuscitation; EMS = emergency medical service (Ambulance Victoria); IQR = interquartile range; SD = standard deviation.

* Excludes emergency medical service‐witnessed events.

Missing data: † 2018 (2.0%); ‡ 367 (0.4%); § 576 (0.6%); ¶ 305 (0.3%).

Box 5 Out‐of‐hospital cardiac arrest event characteristics, Victoria, 2003–2022*

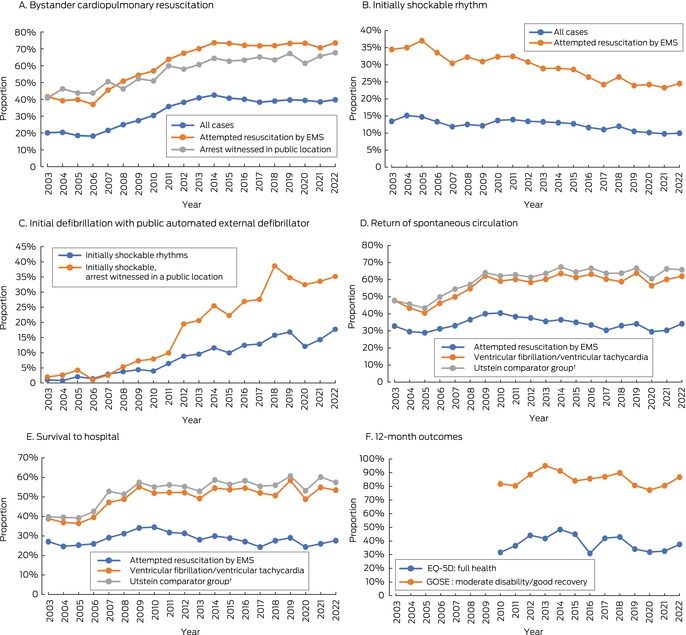

EMS = emergency medical services; GOSE = Glasgow Outcome Scale–Extended.

* Excludes emergency medical service‐witnessed events. The data underlying this graph are included in the Supporting Information, tables 4 to 9.

† Bystander‐witnessed arrests in which the initial cardiac rhythm was ventricular fibrillation or ventricular tachycardia, and resuscitation was attempted by emergency medical services.

Box 6 Characteristics of out‐of‐hospital cardiac arrests in which emergency medical services attempted resuscitation, Victoria, 2003–2022, by two‐year period*

**Table:** 

Characteristic	Overall	2003/2004	2005/2006	2007/2008	2009/2010	2011/2012	2013/2014	2015/2016	2017/2018	2019/2020	2021/2022
Total	43 195	3543	3422	3519	3785	4048	4518	4660	5049	5132	5519
Age (years), mean (SD)^†^	62.9 (20.6)	63.6 (19.7)	62.7 (20.6)	62.7 (21.0)	62.2 (21.1)	62.3 (20.9)	63.4 (20.1)	62.4 (20.9)	62.7 (20.7)	62.7 (20.9)	63.6 (20.2)
Sex (men)^‡^	29 761 (68.9%)	2426 (68.5%)	2417 (70.6%)	2467 (70.2%)	2577 (68.1%)	2792 (69.0%)	3069 (68.0%)	3254 (69.8%)	3458 (68.5%)	3507 (68.4%)	3794 (68.8%)
Metropolitan location	31 420 (72.7%)	2751 (77.7%)	2614 (76.4%)	2740 (77.9%)	2804 (74.1%)	2892 (71.4%)	3208 (71.0%)	3329 (71.4%)	3591 (71.1%)	3592 (70.0%)	3899 (70.7%)
Location											
Private residence	29,936 (69.3%)	2456 (69.3%)	2336 (68.3%)	2431 (69.1%)	2594 (68.5%)	2740 (67.7%)	3121 (69.1%)	3226 (69.2%)	3462 (68.6%)	3615 (70.4%)	3955 (71.7%)
Aged care facility	3299 (7.6%)	209 (5.9%)	216 (6.3%)	256 (7.3%)	292 (7.7%)	287 (7.1%)	390 (8.6%)	377 (8.1%)	442 (8.8%)	423 (8.2%)	407 (7.4%)
Public location	8738 (20.2%)	762 (21.5%)	744 (21.7%)	728 (20.7%)	788 (20.8%)	909 (22.5%)	884 (19.6%)	921 (19.8%)	1015 (20.1%)	959 (18.7%)	1028 (18.6%)
Other	1222 (2.8%)	116 (3.3%)	126 (3.7%)	104 (3.0%)	111 (2.9%)	112 (2.8%)	123 (2.7%)	136 (2.9%)	130 (2.6%)	135 (2.6%)	129 (2.3%)
Precipitating event											
Presumed cardiac	32 772 (75.9%)	2798 (79.0%)	2694 (78.7%)	2772 (78.8%)	2959 (78.2%)	3007 (74.3%)	3392 (75.1%)	3489 (74.9%)	3812 (75.5%)	3819 (74.4%)	4030 (73.0%)
Trauma/hanging	3129 (7.2%)	197 (5.6%)	198 (5.8%)	167 (4.8%)	243 (6.4%)	290 (7.2%)	317 (7.0%)	343 (7.4%)	424 (8.4%)	463 (9.0%)	487 (8.8%)
Overdose/poisoning	1826 (4.2%)	144 (4.1%)	107 (3.1%)	146 (4.2%)	177 (4.7%)	142 (3.5%)	178 (3.9%)	215 (4.6%)	242 (4.8%)	242 (4.7%)	233 (4.2%)
Other	5468 (12.7%)	404 (11.4%)	423 (12.4%)	434 (12.3%)	406 (10.7%)	609 (15.0%)	631 (14.0%)	613 (13.2%)	571 (11.3%)	608 (11.9%)	769 (13.9%)
EMS response time (min), median (IQR)^§^	8.0 (6.1–10.9)	7.0 (6.0–10.0)	8.0 (6.0–10.0)	8.0 (6.3–10.8)	8.2 (6.5–11.1)	8.4 (6.5–11.8)	8.3 (6.4–11.5)	7.8 (6.1–10.8)	7.6 (6.0–10.4)	7.8 (6.2–10.3)	8.2 (6.4–11.2)
Bystander‐witnessed	23 402 (54.2%)	2158 (60.9%)	2093 (61.2%)	1970 (56.0%)	2186 (57.8%)	2281 (56.4%)	2435 (53.9%)	2394 (51.4%)	2505 (49.6%)	2549 (49.7%)	2831 (51.3%)
Bystander CPR	27 217 (63.0%)	1429 (40.3%)	1316 (38.5%)	1696 (48.2%)	2107 (55.7%)	2657 (65.6%)	3250 (71.9%)	3386 (72.7%)	3630 (71.9%)	3762 (73.3%)	3984 (72.2%)
Initial rhythm^¶^											
Ventricular fibrillation/ventricular tachycardia	12 377 (28.8%)	1226 (34.8%)	1201 (35.3%)	1094 (31.4%)	1187 (31.7%)	1272 (31.6%)	1304 (29.0%)	1275 (27.5%)	1273 (25.3%)	1228 (24.1%)	1317 (23.9%)
Pulseless electrical activity	8856 (20.6%)	869 (24.7%)	832 (24.5%)	742 (21.3%)	760 (20.3%)	849 (21.1%)	888 (19.7%)	1018 (21.9%)	1058 (21.1%)	983 (19.3%)	857 (15.6%)
Asystole	20 484 (47.7%)	1370 (38.9%)	1340 (39.4%)	1638 (47.0%)	1792 (47.8%)	1875 (46.6%)	2285 (50.7%)	2340 (50.4%)	2579 (51.3%)	2624 (51.4%)	2641 (48.0%)
Non‐shockable (unspecified)	1248 (2.9%)	61 (1.7%)	29 (0.9%)	14 (0.4%)	11 (0.3%)	25 (0.6%)	27 (0.6%)	8 (0.2%)	117 (2.3%)	266 (5.2%)	690 (12.5%)
Utstein cohort	9547 (22.1%)	929 (26.2%)	928 (27.1%)	829 (23.6%)	941 (24.9%)	990 (24.5%)	1043 (23.1%)	989 (21.2%)	962 (19.1%)	935 (18.2%)	1001 (18.1%)
Initial shock provider**^,§§^											
Paramedics	10 104 (82.2%)	1125 (92.4%)	1095 (92.2%)	951 (88.1%)	1028 (87.3%)	1038 (81.8%)	1.053 (81.0%)	1036 (81.6%)	960 (75.8%)	915 (75.0%)	903 (69.0%)
First responders	1129 (9.2%)	82 (6.7%)	72 (6.1%)	92 (8.5%)	101 (8.6%)	134 (10.6%)	109 (8.4%)	92 (7.2%)	125 (9.9%)	128 (10.5%)	194 (14.8%)
Public access defibrillator	1064 (8.7%)	11 (0.9%)	21 (1.8%)	36 (3.3%)	49 (4.2%)	97 (7.6%)	138 (10.6%)	142 (11.2%)	182 (14.4%)	177 (14.5%)	211 (16.1%)
Return of spontaneous circulation	14 671 (34.0%)	1106 (31.2%)	1029 (30.1%)	1227 (34.9%)	1524 (40.3%)	1536 (37.9%)	1630 (36.1%)	1595 (34.2%)	1600 (31.7%)	1638 (31.9%)	1786 (32.4%)
Pulse on hospital arrival^††^	12 161 (28.3%)	909 (25.8%)	867 (25.6%)	1048 (30.2%)	1292 (34.3%)	1272 (31.6%)	1310 (29.1%)	1301 (28.0%)	1309 (26.0%)	1372 (26.8%)	1481 (26.8%)
Discharged alive^‡‡^	4160 (9.7%)	241 (6.9%)	283 (8.4%)	327 (9.4%)	396 (10.6%)	442 (11.0%)	437 (9.7%)	508 (10.9%)	537 (10.7%)	509 (10.0%)	480 (8.8%)

CPR = cardiopulmonary resuscitation; EMS = emergency medical service; IQR = interquartile range; SD = standard deviation.

* Excludes emergency medical service‐witnessed events.

Missing data: † 113 (0.3%); ‡ 17 (0.04%); § 134 (0.3%); ¶ 230 (0.5%); ** 80 (0.7%); †† 174 (0.4%); ‡‡ 314 (0.7%); §§ Proportions of patients with initial rhythm of ventricular fibrillation/ventricular tachycardia.

#### Initially shockable rhythm and public access defibrillation

The proportion of people with initially shockable rhythms declined from 14.3% in 2003/2004 to 9.9% in 2021/2022 (Box 4, Box 5B); in cases in which EMS personnel attempted resuscitation, this proportion declined from 34.8% to 23.9% (Box 6, Box 5B). The proportion of people with initially shockable rhythms who were first defibrillated with public AEDs increased from 0.9% in 2003/2004 to 16.1% in 2021/2022 (Box 6, Box 5C). The adjusted odds of public access defibrillation in 2022 were higher than 2003 (aOR, 22.2; 95% CI, 9.65–51.2).

#### Emergency medical services response time

The median EMS response time increased across the study period, both overall (from 7.0 [IQR, 6.0–10.0] minutes in 2003/2004 to 9.0 [IQR, 6.7–14.8] minutes in 2021/2022) and for OHCAs in which EMS attempted resuscitation (from 7.0 [IQR, 6.0–10.0] to 8.2 [IQR, 6.4–11.2] minutes).

#### Pre‐hospital return of spontaneous circulation and survival to hospital arrival

In cases in which EMS personnel attempted resuscitation, the proportions of patients who experienced pre‐hospital return of spontaneous circulation or survived to hospital arrival did not change markedly across the study period. In the Utstein comparator group, the proportion of cases with pre‐hospital return of spontaneous circulation increased from 47.8% in 2003 to 65.8% in 2022, and that of survival to hospital arrival increased from 39.8% to 57.4%, despite minor reductions in 2020 (Box 5D, Box 5E, Box 6).

#### Twelve‐month outcomes

Among the 4452 adults discharged from hospital alive during 1 January 2010 – 30 June 2022, 4155 (93.3%) were alive at twelve months. Of 3249 people (78.2%) who responded to 12‐month follow‐up enquiries, 2754 (85.3%) had Glasgow Outcome Scale–Extended scores indicating good recovery or moderate disability, and 1218 people (38.5%) reported full health according to the EQ‐5D (Box 5F).

## Discussion

In our analysis of OHCA registry data for Victoria, 2003–2022, we found that survival to hospital discharge increased threefold in the Utstein comparator group over the 20‐year study period, and fourfold during 2003–2019. The odds of bystanders providing CPR increased 4.5‐fold during 2003–2022, although the COVID‐19 pandemic was associated with a 37% reduction in the odds of survival.

In our adjusted model, the largest survival differences relative to 2012 were associated with the public launch of the AED registry and GoodSAM in 2018, and the start of the Ambulance Victoria resuscitation quality improvement program in 2019. Volunteer responder smartphone applications increase the provision of bystander interventions for people with OHCA,^9^, ^10^ but their impact on survival is unclear.^19^ We cannot attribute the higher adjusted odds ratios in 2018 and 2019 to the smartphone application alone, as we did not assess volunteer arrival order or interventions. However, the integrated AED registry enabled the communication of public AED location information to responders, and the proportion of cases in which defibrillation was provided with public AEDs also increased in 2018. The effectiveness of the GoodSAM application should be investigated further.

We found that the proportions of cases with survival to hospital arrival and pre‐hospital return of spontaneous circulation were larger in 2021 and 2022 than in 2020, and similar to those of 2019. However, the proportion of people who survived to hospital discharge did not return to pre‐COVID‐19 levels, despite similar OHCA characteristics before and after the pandemic. The factors underlying these differences require further review, including hospital‐related factors. The COVID‐19 pandemic affected health care systems everywhere; longer emergency department waiting times and lengths of stay^20^ and staff burnout^21^ were reported in Victoria. The impact of these factors on OHCA outcomes warrants further investigation.

The higher OHCA incidence in 2022 could also be related to the COVID‐19 pandemic in terms of mortality displacement. As COVID‐19‐related restrictions in Victoria may have protected older and frail people; after restrictions were lifted in 2022, their mortality risk may have increased. The age‐standardised death rate in Australia was indeed lower in 2020–21 than in 2015–19,^22^ and excess mortality in Victoria was 13% higher in 2022 than predicted.^23^

The proportion of OHCA events in which CPR was provided by bystanders increased during 2002–2014, but plateaued during 2014–2022. As EMS response times are increasing because of the ever‐rising demand for their assistance, and the proportion of OHCA cases with initially shockable rhythms is declining, boosting the responsiveness of community members to OHCA events is critical. Campaigns such as World Restart a Heart Day^24^ and local Heart Safe Communities^25^ aim to build community knowledge about OHCA and encourage bystander resuscitation. Further, the Kids Save Lives program, recently expanded in Victoria, aims to teach children three simple steps for saving a life (Call, Push, Shock).^26^ The First Responder Shock Trial^27^ is also underway in our region, equipping frequent GoodSAM responders with ultraportable AEDs. Other initiatives for improving survival could include expanding the firefighter first responder program, and AED drone delivery,^28^ as well as fostering an EMS culture of excellence.^29^ The VACAR will be pivotal in informing and evaluating such initiatives.

Finally, the VACAR is one of the few cardiac arrest registries to routinely capture data on the health‐related quality of life and functional recovery of people who survive OHCA. Ours is one of the first studies to examine long term trends in 12‐month outcomes; unfortunately, we did not find significant improvements in outcomes during the study period. Reductions in the proportion of cases with favourable outcomes during 2019–2021 may be related to the COVID‐19 pandemic and associated restrictions, but further investigations are needed.

### Limitations

Our study was limited by its retrospective nature. As the VACAR does not capture OHCA events not attended by EMS in Victoria (eg, cases in which death is expected), we will have underestimated the incidence of OHCA. The VACAR predominantly collects pre‐hospital arrest factors; changes in hospital‐related factors that influence survival to discharge could not be evaluated. Fluctuations in health‐related quality of life and functional recovery outcomes may be related to changes in the interview staff.

### Conclusion

Survival to hospital discharge of people who experienced bystander‐witnessed OHCA with initially shockable cardiac rhythms increased threefold in Victoria during 2003–2022, and fourfold during 2003–2019. The proportion of cases in which bystanders attempted CPR or defibrillation also grew significantly. However, survival gains were diminished during the COVID‐19 pandemic, and new strategies are needed to improve outcomes.

## Competing interests

No relevant disclosures.

## Data sharing

Access to the data and analysis files is permitted only with the explicit permission of the approving human research ethics committee and the data custodians.

## Supporting information


Supplementary results
