# Supplementary material for: Out‐of‐hospital cardiac arrests in Victoria, 2003–2022: retrospective analysis of Victorian Ambulance Cardiac Arrest Registry data
Source: Med J Aust. 2024 Nov 18;221(11):603–11. doi: 10.5694/mja2.52532 (PMC11625529; doi:10.5694/mja2.52532)
Supplement: Supplementary file 1 — Supplementary results [file MJA2-221-603-s001.pdf]

MJA
The Medical Journal of Australia

Supporting Information

Supplementary results

**This appendix was part of the submitted manuscript and has been peer reviewed.
It is posted as supplied by the authors.**

Appendix to: Nehme E, Anderson D, Salathiel R, et al. Out-of-hospital cardiac arrests in Victoria, 2003–2022: retrospective analysis of Victorian Ambulance Cardiac Arrest Registry data. *Med J Aust* 2024; doi: 10.5694/mja2.52532.

Table 1. Crude and age-standardised incidence of out-of-hospital cardiac arrest, Victoria, 2003–2022*

	Incidence (per 100,000 population)	
	Crude	Age-standardised
**2003**	90.6	89.1
**2004**	83.0	80.9
**2005**	85.4	82.6
**2006**	82.9	79.5
**2007**	83.2	79.2
**2008**	84.6	79.8
**2009**	86.4	81.2
**2010**	81.4	76.0
**2011**	82.8	76.6
**2012**	82.6	76.2
**2013**	83.2	75.9
**2014**	87.3	79.1
**2015**	85.2	77.3
**2016**	88.5	80.5
**2017**	89.0	80.5
**2018**	87.9	79.0
**2019**	93.0	83.1
**2020**	92.5	81.5
**2021**	97.3	83.8
**2022**	107.4	91.2

* Excludes emergency medical service-witnessed events.

Table 2. Survival to hospital discharge after out-of-hospital cardiac arrest, Victoria, 2003–2022*

	All attempted resuscitation cases	All cases with initial ventricular fibrillation or ventricular tachycardia and attempted resuscitation by emergency medical services	Utstein comparator group†
**2003**	109/1723 (6.3%)	82/588 (13.9%)	68/445 (15.3%)
**2004**	132/1779 (7.4%)	103/617 (16.7%)	86/466 (18.5%)
**2005**	137/1699 (8.1%)	107/626 (17.1%)	91/484 (18.8%)
**2006**	146 /1680 (8.7%)	113/556 (20.3%)	97/429 (22.6%)
**2007**	147/1703 (8.6%)	107/509 (21.0%)	92/366 (25.1%)
**2008**	180/1765 (10.2%)	134/552 (24.3%)	109/433 (25.2%)
**2009**	198/1854 (10.7%)	158/565 (28.0%)	140/457 (30.6%)
**2010**	198/1889 (10.5%)	163/596 (27.3%)	135/462 (29.2%)
**2011**	215/1983 (10.8%)	183/637 (28.7%)	154/479 (32.2%)
**2012**	227/2053 (11.1%)	188/629 (29.9%)	166/506 (32.8%)
**2013**	202/2180 (9.3%)	168/625 (26.9%)	151/500 (30.2%)
**2014**	235/2326 (10.1%)	193/669 (28.8%)	174/534 (32.6%)
**2015**	254/2270 (11.2%)	201/643 (31.3%)	166/492 (33.7%)
**2016**	254/2375 (10.7%)	209/625 (33.4%)	179/490 (36.5%)
**2017**	257/2519 (10.2%)	200/600 (33.3%)	167/453 (36.9%)
**2018**	280/2505 (11.2%)	221/657 (33.6%)	195/496 (39.3%)
**2019**	303/2632 (11.5%)	240/626 (38.3%)	207/484 (42.8%)
**2020**	206/2481 (8.3%)	162/596 (27.2%)	141/449 (31.4%)
**2021**	215/2626 (8.2%)	179/606 (29.5%)	155/452 (34.3%)
**2022**	265/2839 (9.3%)	216/690 (31.3%)	187/528 (35.4%)

* Excludes emergency medical service-witnessed events.

† Bystander-witnessed arrests in which the initial cardiac rhythm was ventricular fibrillation or ventricular tachycardia, and resuscitation was attempted by emergency medical services.

Table 3. Survival to hospital discharge after out-of-hospital cardiac arrest, Victoria, 2003–2022, for cases in the Utstein comparator group*

	Odds ratio (95% confidence interval)
**2003**	0.39 (0.28–0.54)
**2004**	0.51 (0.37–0.69)
**2005**	0.51 (0.38–0.70)
**2006**	0.55 (0.41–0.75)
**2007**	0.66 (0.48–0.90)
**2008**	0.69 (0.51–0.94)
**2009**	0.96 (0.72–1.28)
**2010**	0.86 (0.64–1.14)
**2011**	1.00 (0.75–1.32)
**2012**	1
**2013**	0.93 (0.70–1.23)
**2014**	1.14 (0.86–1.49)
**2015**	1.09 (0.83–1.45)
**2016**	1.23 (0.93–1.62)
**2017**	1.22 (0.92–1.61)
**2018**	1.37 (1.04–1.80)
**2019**	1.68 (1.28–2.21)
**2020**	1.06 (0.80–1.42)
**2021**	1.18 (0.89–1.56)
**2022**	1.19 (0.90–1.56)

* Adjusted for study year, age, gender, aetiology (presumed cardiac v trauma or hanging, overdose or poisoning, or other), arrest location (public location v private residence, aged care facility, or other), and arrest geographic location (metropolitan v regional).

Table 4. Out-of-hospital cardiac arrest event characteristics, Victoria, 2003–2022.*
Bystander cardiopulmonary resuscitation

	All cases	Attempted resuscitation by emergency medical services	Arrest witnessed in a public location
**2003**	898/4457 (20.1%)	721/1737 (41.5%)	184/451 (40.8%)
**2004**	860/4213 (20.4%)	708/1806 (39.2%)	202/437 (46.2%)
**2005**	803/4351 (18.5%)	683/1712 (39.9%)	197/450 (43.8%)
**2006**	783/4312 (18.2%)	633/1710 (37.0%)	179/409 (43.8%)
**2007**	956/4449 (21.5%)	788/1732 (45.5%)	183/362 (50.6%)
**2008**	1143/4570 (25.0%)	908/1787 (50.8%)	185/400 (46.3%)
**2009**	1307/4773 (27.4%)	1019/1873 (54.4%)	233/446 (52.2%)
**2010**	1382/4540 (30.4%)	1088/1912 (56.9%)	239/469 (51.0%)
**2011**	1659/4644 (35.7%)	1268/1987 (63.8%)	284/474 (59.9%)
**2012**	1817/4747 (38.3%)	1389/2061 (67.4%)	299/516 (57.9%)
**2013**	1969/4817 (40.9%)	1534/2187 (70.1%)	290/478 (60.7%)
**2014**	2199/5173 (42.5%)	1716/2331 (73.6%)	311/483 (64.4%)
**2015**	2100/5162 (40.7%)	1670/2279 (73.3%)	294/469 (62.7%)
**2016**	2173/5432 (40.0%)	1716/2381 (72.1%)	313/494 (63.4%)
**2017**	2143/5593 (38.3%)	1821/2534 (71.9%)	321/492 (65.2%)
**2018**	2194/5623 (39.0%)	1809/2515 (71.9%)	291/459 (63.4%)
**2019**	2419/6092 (39.7%)	1937/2643 (73.3%)	329/489 (67.3%)
**2020**	2410/6113 (39.4%)	1825/2489 (73.3%)	207/337 (61.4%)
**2021**	2455/6374 (38.5%)	1877/2655 (70.7%)	270/411 (65.7%)
**2022**	2847/7157 (39.8%)	2107/2864 (73.6%)	336/496 (67.7%)

* Excludes emergency medical service-witnessed events.

Table 5. Out-of-hospital cardiac arrest event characteristics, Victoria, 2003–2022*. Initially shockable rhythm

	All cases	Attempted resuscitation by emergency medical services
**2003**	598/4445 (13.5%)	596/1728 (34.5%)
**2004**	636/4197 (15.2%)	630/1798 (35.0%)
**2005**	639/4334 (14.7%)	632/1706 (37.1%)
**2006**	572/4291 (13.3%)	569/1696 (33.6%)
**2007**	526/4424 (11.9%)	523/1718 (30.4%)
**2008**	571/4549 (12.6%)	571/1770 (32.3%)
**2009**	578/4758 (12.2%)	575/1859 (30.9%)
**2010**	620/4519 (13.7%)	612/1891 (32.4%)
**2011**	644/4618 (14.0%)	639/1968 (32.5%)
**2012**	638/4739 (13.5%)	633/2053 (30.8%)
**2013**	640/4809 (13.3%)	631/2181 (28.9%)
**2014**	675/5163 (13.1%)	673/2323 (29.0%)
**2015**	656/5147 (12.8%)	648/2264 (28.6%)
**2016**	633/5428 (11.7%)	627/2377 (26.4%)
**2017**	615/5573 (11.0%)	610/2519 (24.2%)
**2018**	674/5615 (12.0%)	663/2508 (26.4%)
**2019**	639/6072 (10.5%)	628/2625 (23.9%)
**2020**	618/6091 (10.2%)	600/2476 (24.2%)
**2021**	624/6367 (9.8%)	617/2649 (23.3%)
**2022**	713/7148 (10.0%)	700/2856 (24.5%)

* Excludes emergency medical service-witnessed events.

Table 6. Out-of-hospital cardiac arrest event characteristics, Victoria, 2003–2022*. Initial defibrillation with public automated external defibrillator

	All initially shockable rhythms	Initially shockable rhythm, arrest witnessed in a public location
**2003**	6/592 (1.0%)	3/152 (2.0%)
**2004**	5/626 (0.8%)	5/189 (2.7%)
**2005**	13/624 (2.1%)	7/166 (4.2%)
**2006**	8/564 (1.4%)	2/184 (1.1%)
**2007**	15/514 (2.9%)	4/154 (2.6%)
**2008**	21/565 (3.7%)	9/169 (5.3%)
**2009**	25/571 (4.4%)	14/191 (7.3%)
**2010**	24/607 (4.0%)	14/177 (7.9%)
**2011**	41/637 (6.4%)	20/202 (9.9%)
**2012**	56/632 (8.9%)	40/205 (19.5%)
**2013**	60/628 (9.6%)	39/189 (20.6%)
**2014**	78/672 (11.6%)	52/204 (25.5%)
**2015**	64/645 (9.9%)	43/193 (22.3%)
**2016**	78/625 (12.5%)	52/193 (26.9%)
**2017**	78/607 (12.9%)	53/192 (27.6%)
**2018**	104/660 (15.8%)	75/194 (38.7%)
**2019**	105/624 (16.8%)	73/210 (34.8%)
**2020**	72/596 (12.1%)	38/117 (32.5%)
**2021**	88/615 (14.3%)	52/155 (33.6%)
**2022**	123/693 (17.8%)	70/199 (35.2%)

* Excludes emergency medical service-witnessed events. Includes patients with an attempted resuscitation by emergency medical services.

Table 7. Out-of-hospital cardiac arrest event characteristics, Victoria, 2003–2022*. Return of spontaneous circulation

	Attempted resuscitation by emergency medical services	Cases with initial ventricular fibrillation or ventricular tachycardia and attempted resuscitation by emergency medical services	Utstein comparator group†
**2003**	571/1,737 (32.9%)	285/596 (47.8%)	216/452 (47.8%)
**2004**	535/1,806 (29.6%)	273/630 (43.3%)	218/477 (45.7%)
**2005**	495/1,712 (28.9%)	256/632 (40.5%)	212/489 (43.4%)
**2006**	534/1,710 (31.2%)	263/569 (46.2%)	219/439 (49.9%)
**2007**	573/1,732 (33.1%)	261/523 (49.9%)	206/378 (54.5%)
**2008**	654/1,787 (36.6%)	313/571 (54.8%)	258/451 (57.2%)
**2009**	751/1,873 (40.1%)	358/575 (62.3%)	298/465 (64.1%)
**2010**	773/1,912 (40.4%)	362/612 (59.2%)	296/476 (62.2%)
**2011**	761/1,987 (38.3%)	385/639 (60.3%)	302/480 (62.9%)
**2012**	775/2,061 (37.6%)	370/633 (58.5%)	313/510 (61.4%)
**2013**	778/2,187 (35.6%)	380/631 (60.2%)	322/506 (63.6%)
**2014**	852/2,331 (36.6%)	428/673 (63.6%)	362/537 (67.4%)
**2015**	798/2,279 (35.0%)	397/648 (61.3%)	320/497 (64.4%)
**2016**	797/2,381 (33.5%)	396/627 (63.2%)	328/492 (66.7%)
**2017**	769/2,534 (30.4%)	368/610 (60.3%)	293/460 (63.7%)
**2018**	831/2,515 (33.0%)	390/663 (58.8%)	320/502 (63.8%)
**2019**	903/2,643 (34.2%)	401/628 (63.9%)	323/484 (66.7%)
**2020**	735/2,489 (29.5%)	339/600 (56.5%)	273/451 (60.5%)
**2021**	806/2,655 (30.4%)	371/617 (60.1%)	307/463 (66.3%)
**2022**	980/2,864 (34.2%)	434/700 (62.0%)	354/538 (65.8%)

* Excludes emergency medical service-witnessed events.
† Bystander-witnessed arrests in which the initial cardiac rhythm was ventricular fibrillation or ventricular tachycardia, and resuscitation was attempted by emergency medical services.

Table 8. Out-of-hospital cardiac arrest event characteristics, Victoria, 2003–2022*. Survival to hospital arrival

	Attempted resuscitation by emergency medical services	All cases with initial ventricular fibrillation or ventricular tachycardia and attempted resuscitation by emergency medical services	Utstein comparator group†
**2003**	467/1,726 (27.1%)	231/594 (38.9%)	179/450 (39.8%)
**2004**	442/1,793 (24.7%)	230/623 (36.9%)	187/473 (39.5%)
**2005**	428/1,692 (25.3%)	227/623 (36.4%)	189/482 (39.2%)
**2006**	439/1,691 (26.0%)	221/559 (39.5%)	183/430 (42.6%)
**2007**	498/1,710 (29.1%)	243/515 (47.2%)	196/371 (52.8%)
**2008**	550/1,763 (31.2%)	274/561 (48.8%)	227/442 (51.4%)
**2009**	635/1,860 (34.1%)	316/573 (55.2%)	266/463 (57.5%)
**2010**	657/1,904 (34.5%)	317/610 (52.0%)	261/474 (55.1%)
**2011**	630/1,981 (31.8%)	333/637 (52.3%)	269/479 (56.2%)
**2012**	642/2,051 (31.3%)	329/630 (52.2%)	281/508 (55.3%)
**2013**	614/2,185 (28.1%)	310/630 (49.2%)	267/505 (52.9%)
**2014**	696/2,325 (29.9%)	366/671 (54.6%)	314/535 (58.7%)
**2015**	657/2,275 (28.9%)	348/648 (53.7%)	280/497 (56.3%)
**2016**	644/2,377 (27.1%)	342/627 (54.6%)	287/492 (58.3%)
**2017**	615/2,528 (24.3%)	317/609 (52.1%)	255/460 (55.4%)
**2018**	694/2,514 (27.6%)	336/663 (50.7%)	281/502 (56.0%)
**2019**	766/2,640 (29.0%)	367/628 (58.4%)	294/484 (60.7%)
**2020**	606/2,489 (24.4%)	293/600 (48.8%)	240/451 (53.2%)
**2021**	690/2,653 (26.0%)	338/616 (54.9%)	278/462 (60.2%)
**2022**	791/2,864 (27.6%)	374/700 (53.4%)	309/538 (57.4%)

* Excludes emergency medical service-witnessed events.
† Bystander-witnessed arrests in which the initial cardiac rhythm was ventricular fibrillation or ventricular tachycardia, and resuscitation was attempted by emergency medical services.

Table 9. Out-of-hospital cardiac arrest event characteristics, Victoria, 2003–2022*. Twelve-month outcomes

	EQ-5D index: full health	Glasgow Outcomes Scale-Extended: moderate disability or good recovery
**2010**	73/230 (31.7%)	189/231 (81.8%)
**2011**	81/222 (36.5%)	181/225 (80.4%)
**2012**	109/247 (44.1%)	218/246 (88.6%)
**2013**	93/222 (41.9%)	214/225 (95.1%)
**2014**	124/256 (48.4%)	234/256 (91.4%)
**2015**	112/249 (45.0%)	242/288 (84.0%)
**2016**	87/281 (31.0%)	249/291 (85.6%)
**2017**	109/259 (42.1%)	234/269 (87.0%)
**2018**	131/305 (43.0%)	275/306 (89.9%)
**2019**	101/296 (34.1%)	239/296 (80.7%)
**2020**	76/238 (31.9%)	184/238 (77.3%)
**2021**	77/236 (32.6%)	190/236 (80.5%)
**2022**	45/120 (37.5%)	105/121 (86.8%)

* Excludes emergency medical service-witnessed events.
